# The *Streptococcus pneumoniae pezAT* Toxin–Antitoxin System Reduces β-Lactam Resistance and Genetic Competence

**DOI:** 10.3389/fmicb.2016.01322

**Published:** 2016-08-25

**Authors:** Wai T. Chan, Manuel Espinosa

**Affiliations:** Bacterial Gene Expression and Gene Transfer, Molecular Microbiology and Infectious Biology, Centro de Investigaciones Biológicas, Consejo Superior de Investigaciones CientíficasMadrid, Spain

**Keywords:** *Streptococcus pneumoniae*, toxin–antitoxin, *pezAT*, antibiotic resistance, genetic competence, genetic transformation

## Abstract

Chromosomally encoded Type II Toxin–Antitoxin operons are ubiquitous in bacteria and archaea. Antitoxins neutralize the toxic effect of cognate Toxins by protein–protein interactions and sequestering the active residues of the Toxin. Toxins target essential bacterial processes, mostly translation and replication. However, one class apart is constituted by the PezAT pair because the PezT toxin target cell wall biosynthesis. Here, we have examined the role of the *pezAT* toxin–antitoxin genes in its natural host, the pathogenic bacterium *Streptococcus pneumonia*e. The *pezAT* operon on Pneumococcal Pathogenicity Island 1 was deleted from strain R6 and its phenotypic traits were compared with those of the wild type. The mutant cells formed shorter chains during exponential phase, leading to increased colony-forming units. At stationary phase, the mutant was more resilient to lysis. Importantly, the mutant exhibited higher resistance to antibiotics targeting cell walls (β-lactams), but not to antibiotics acting at other levels. In addition, the mutants also showed enhanced genetic competence. We suggest that PezAT participates in a subtle equilibrium between loss of functions (resistance to β-lactams and genetic competence) and gain of other traits (virulence).

## Introduction

*Streptococcus pneumoniae* (the pneumococcus) is a leading cause of many infections, mainly pneumococcal pneumonia, meningitis (up to 50% of the reported cases), sepsis, otitis media, and other minor infections. The estimate rate of mortality is about 1.4 million per year, among them children below 5 years and elderly people (Mandell et al., 2007). Management of pneumococcal infections takes into account the vaccination programs, although these have led to selection of serotypes for which vaccination is not available (Meichtry et al., 2014). Antibiotic treatments for pneumococcal diseases have traditionally been penicillin, but increased antibiotic resistance due to selection of strains with altered penicillin-binding proteins have been reported (Chambers, 1999). Approaches to tackle pneumococcal diseases other than employment of antibiotics have been proposed (reviewed in Chan et al., 2015), and promising, more general, strategies involve the exploitation of the pneumococcal TA genes as likely candidates for drug development (Mutschler and Meinhart, 2011; Shapiro, 2013; Chan et al., 2015).

Bacterial TAs have become the focus of attention of basic and applied research as they are ubiquitous (most of bacteria have them), redundant (up to 88 copies per bacterial chromosome), and they may constitute good targets for drug development (Chan et al., 2015). In general, most TAs are built as a single transcriptional unit where the antitoxin gene precedes the one encoding the toxin. The most frequent and best studied class of TA operons is those pertaining to the Type II, in which the two elements are proteins. Under steady-state conditions, the TA proteins form a complex that self-regulate and that is harmless to the bacteria. However, under stressful conditions, such as nutritional stress, the antitoxin (which is more labile) is more readily degraded by host proteases, thus releasing a free stable toxin protein that will act on its cellular target. TAs constitute an intriguing example of acquisition of genetic information through horizontal gene transfer followed by manipulation/domestication by the host bacterial population to serve as relevant players in their lifestyle. When TAs are located in mobile elements, they act as systems that ensure their stable inheritance (Hayes, 2003; Guglielmini et al., 2008). However, once integrated into the bacterial chromosome, they seem to have been domesticated as to perform a number of different functions related to the bacterial physiology, namely response to stressful conditions by causing temporal cell growth arrest, mediators of programmed cell death, or persistence as a response to antibiotic challenges (Engelberg-Kulka et al., 2004; Gerdes et al., 2005; Díaz-Orejas et al., 2010; Chan et al., 2012; Gerdes and Maisonneuve, 2012; Gerdes, 2013).

Most of the reported toxins of Type II TA genes target translation (Christensen and Gerdes, 2003) or chromosomal replication (Couturier et al., 1998) of bacterial cells at different stages. A distinct class of TAs has been discovered in streptococci: the Epsilon-Zeta pair found on the broad host-range plasmid pSM19035 of *Streptococcus pyogenes* and its homolog, the PezAT, found on the chromosome of *S. pneumoniae* (Camacho et al., 2002; Khoo et al., 2007; Mutschler and Meinhart, 2011). PezT and Zeta toxins, however, are unique because they are the only known TAs that target cell walls. The toxin catalyzes the phosphorylation, dependent of ATP, of the UNAG, thus converting it into UNAG-3′-P (Mutschler et al., 2011). Since UNAG is the universal precursor of the sugar backbone of the peptidoglycan macromolecules that integrates bacterial cell walls, the PezT-mediated UNAG-3′-P product would hinder the activity of MurA by acting as a competitive inhibitor of UNAG. The MurA enzyme is essential at catalyzing the initial step of bacterial peptidoglycan biosynthesis. Activity of PezT was shown to trigger autolysis in *Escherichia coli*, which was counteracted by the presence of the cognate antitoxin PezA (Mutschler et al., 2011). Bioinformatics search in NCBI databases showed that *pezAT* was present in two-thirds of the 48 pneumococcal strains examined, and some of the pneumococcal stains harbor two copies of the *pezAT* operon (Chan et al., 2012, 2016; Rocker and Meinhart, 2016). These findings agree with a previous study, which showed that 33% of 26 capsular serotypes of *S. pneumoniae* lacked *pezT* (Brown et al., 2004).

Though *pezAT* seems to be not essential in virulence, disruption of *pezT* did impair virulence in a mouse model, indicating it may modulate virulence of pneumococcal strains that carry the operon (Brown et al., 2004). Further, the *pezAT* genes were found within the PPI1, which is a putative mobile (Wyres et al., 2013) variable region (also a hotspot for recombination) present in highly virulent isolates but not in non-invasive and intermediate-virulent strains (Harvey et al., 2011). Besides PPI1, a copy of the *pezAT* operon was also discovered within a putative pneumococcal integrative and conjugative element Tn*5253* (Chan et al., 2014; Iannelli et al., 2014). Experimental evidence suggested that *pezAT* may play a role in stabilizing the mobile elements within the pneumococcal host (Chan et al., 2014; Iannelli et al., 2014). This suggestion coincided with a study in which a homolog of *pezA*T found in *Streptococcus suis* (termed *sezAT*) was shown to be crucial for inheritance of the Pathogenicity Island (SsPI-1) during cell division (Yao et al., 2015).

In the present work we have investigated the influence of the *pezAT* operon in the pneumococcal lifestyle related to the cell-wall integrity of the bacterium (the target of toxin PezT), like resistance to β-lactam antibiotics, cell morphology, and genetic competence. We chose the R6 strain because it is well-known and harbors only three known TAs, namely RelBE2, YefM-YoeB, and PezAT (Chan et al., 2012, 2014). Whereas the two former TA operons encode toxins that act as RNases (Christensen and Gerdes, 2003; Chan et al., 2011), the *pezAT* operon targets the pneumococcal cell wall, making it an interesting system to be studied in their natural host rather than on a heterologous one. Thus, we replaced the single copy of the *pezAT* operon of strain R6 by a gene cassette harboring a kanamycin-resistance selective marker, and we compared several phenotypic traits of this mutant stain with the *wt*. We have found that the *wt* strain was more prone to lysis than the mutated isogenic strain. Further, the strain devoid of the *pezAT* operon showed increased resistance to β-lactam antibiotics and enhanced acquisition of transforming DNA.

## Materials and Methods

### Bacterial Strains, Growth Condition, and DNA Manipulations

*Streptococcus pneumoniae* R6, *wt* (Hoskins et al., 2001; Tettelin et al., 2001) and its derivatives were usually grown in AGCH medium (Lacks, 1968) with 0.3% sucrose and 0.2% yeast extract at 37°C. When necessary, the medium was supplemented with kanamycin (250 μg/ml), chloramphenicol (1 or 3 μg/ml for cells harboring plasmid pC194r (del Solar and Espinosa, 1992), or streptomycin (100 μg/ml).

DNA manipulations and other molecular biology techniques were performed according to standard protocols (Sambrook and Russel, 2001) or manufacturers’ instructions when commercial kits were used. Genomic DNA was isolated with Bacterial Genomic DNA Isolation Kit (Norgen Biotech, Corp.); whereas plasmid DNA was extracted with High Pure Plasmid Isolation kit (Roche), but the protocols were slightly modified to account for the low G+C content of the pneumococcal genome (Ruiz-Cruz et al., 2010). DNA fragments or PCR-amplified DNA-products were purified with QIAquick Gel Extraction Kit (Qiagen). For DNA sequence verification, samples were sent for automated Sanger sequencing in Secugen S.L., Centro de Investigaciones Biológicas, CSIC, Madrid, and then analyzed by the BioEdit Sequence Alignment Editor version 7.0.4.1 (Hall, 1999).

### Gene Replacement, Gene Insertion, and Constructions of Recombinant Plasmids

#### *S. pneumoniae* R6ΔPezAT

The operon *pezAT* of R6*wt* strain was replaced by the gene encoding resistance to kanamycin from plasmid pR410 (Sung et al., 2001). To construct this mutant, the kanamycin-encoding gene (1073 bp) was amplified using primer pair kan-F/kan-R, whereas regions flanking *pezAT* from the genome of *S. pneumoniae* R6*wt*, i.e., pezATup (550 bp) and pezATdown (553 bp), were amplified using primer pairs pezATup-F/pezATup-R and pezATdown-F/pezATdown-R, respectively (**Supplementary Table S1**). The three PCR-amplified products were digested with *Sac*I and/or *Spe*I (New England Biolabs) and ligated with T4 DNA Ligase (New England Biolabs). The 2148 bp fused product was used to transform *S. pneumoniae* R6*wt* and plated on AGCH agar supplemented with kanamycin. Gene replacement was confirmed by determination of the nucleotide sequence of the entire region in three randomly chosen transformants.

#### *S. pneumoniae* R6luc and R6ΔPezATluc

*Streptococcus pneumoniae* R6luc and R6ΔPezATluc strains were constructed by inserting one copy of the *luc* gene (encoding luciferase) placed under the control of the promoter of the *ssbB* gene as follows: the genomic DNA of *S. pneumoniae* R895 [a gift from J. P. Claverys (Chastanet et al., 2001)] which contains a transcriptional fusion of the promoter of the *ssbB* gene and the *Photinus pyralis luc* gene (that encodes firefly luciferase) followed by a chloramphenicol resistant gene, was used to transform first the strain R6*wt*. Selection was done by spreading the transformants on AGCH agar plates supplemented with chloramphenicol. The recombinant genomic DNA of one colony that exhibited chloramphenicol-resistance was extracted, and the region was sequenced. The entire transcriptional fusion cassette (5092 bp) was then amplified with primer pair ssbB′luc-F/ssbB′luc-R (**Supplementary Table S1**). The amplified DNA was used to transform competent *S. pneumoniae* R6*wt* and *S. pneumoniae* R6ΔPezAT strains and selected as above. The integrity of the construction was verified by determination of the nucleotide sequence of the entire region in four randomly chosen colonies.

#### pC194rPezAT

The region encoding the *pezAT* operon, including its upstream promoter (1737 bp) from *S. pneumoniae* R6*wt* was amplified with primer pair pezAT-F/pezAT-R (**Supplementary Table S1**). The amplified fragments were digested with *Hin*dIII, and then ligated to *Hin*dIII-digested plasmid pC194r (a low copy number derivative of plasmid pC194; del Solar and Espinosa, 1992). The recombinant plasmid DNA obtained was used to transform strain R6ΔPezAT and plated on AGCH agar supplemented with chloramphenicol. This strain was used for complementation assays. Six transformants were randomly selected, their plasmid DNA was extracted, and the integrity of the constructions were verified by determination of the nucleotide sequences.

### Assessment of Growth, Colony-Forming Unit (cfu), Morphology, and Phase Variation

To determine the growth patterns and CFU of the *S. pneumoniae* R6*wt* and R6ΔPezAT mutant, cells were grown overnight until OD_650_∼0.3, and then diluted to OD_650_∼0.03. Cells were then allowed to continue growing in fresh medium at 37°C without shaking. OD_650_ were take every 30 min to assess growth patterns and samples were taken at various OD_650_ (0.2, 0.4, 0.6, and 0.8) and then plated on AGCH 1% agar to assess CFU/ml. Samples were also taken at OD_650_∼0.3 and 0.6 for examination under phase-contrast microscope (Olympus CKX41). Pictures were taken at 100× magnifications, 200 ms.

Phase variation analyses were done as reported (Weiser et al., 1996) with slight modifications. In brief, *S. pneumoniae* R6*wt* and R6ΔPezAT were grown until OD_650_∼0.3. Cells were then diluted and plated on tryptic soy (Conda Pronadisa) plates containing 1% agar onto which 100 μl of catalase (5,000 U/ml; Calbiochem) was added. Cells were grown at 37°C overnight in a 5% CO_2_ incubator. Colony morphology was observed under stereo microscope (Leica).

### Oxidative Stress

Oxidative stress assays were done as reported (Bortoni et al., 2009) with small modifications: *S. pneumoniae* R6*wt* and R6ΔPezAT strains were grown as above until OD_650_∼0.3. For each strain, 1 ml of cells were harvested and resuspended in 0.5 ml 1× PBS (pH 7.0). The cells (50 μl) were mixed with an equal volume of H_2_O_2_ (Merck), to give a final concentration of either 20 mM or 40 mM H_2_O_2_, followed by incubation at 37°C, 20 min. The control mixtures contained cells and 1× PBS (pH 7.0). Serial dilutions were made and the cells were plated on AGCH agar and incubated at 37°C, 15 h. Colonies were counted and the results were represented as percent of survival relative to the control. These assays were repeated four times and the average and standard deviation were calculated.

### Resazurin Microtiter Assay (REMA) Plates

We used resazurin as color indicator to measure the MIC of *S. pneumoniae* R6*wt* and R6ΔPezAT mutant strains. Resazurin is a blue non-fluorescent dye that can be converted to the pink fluorescent dye, resorufin, by metabolically active cells (Tizzard et al., 2006; Mania et al., 2010; Khalifa et al., 2013), a method that has been used for *S. pneumoniae* (Patel et al., 2011; Vandevelde et al., 2014). The color changes can be also measured by light absorbance at 570 nm for resorufin or 600 nm for resazurin. MICs of various antibiotics (ampicillin, benzetacil, levofloxacin, and streptomycin) were determined by using twofold serial dilution method. Briefly, the cells were grown at 37°C until OD_650_∼0.3. Cells were diluted to 1:100 into pre-warmed fresh medium and 100 μl were added into a flat-bottom BD Falcon 96-Well Cell Culture Plate. Different amounts of antibiotics and 200 μM resazurin were added to each well. The mixtures were incubated at 37°C and the color changes were measured at 600 nm every hour by employment of a Varioskan Flash Multimode Reader (Thermo Scientific). These assays were repeated at three times and the mean values and the standard deviations were calculated. *t*-test (with *p*-value < 0.05) was used to evaluate the significant differences of the results. The results were validated by the use of standard MIC determinations on microtiter plates (Andrews, 2001), without resazurin but determining the number of colonies formed by cultures grown in the absence or in the presence of ampicillin.

### Transformability Assays

*Streptococcus pneumoniae* strains R6*wt* and R6ΔPezAT were made competent by preparing cultures from stocks and diluting 1:1,000. Cells were grown at 37°C, and when an OD_650_∼0.3 was reached, two 1:40 successive dilutions were made until OD_650_∼0.3 was reached again. Cultures received 10% glycerol; aliquots of 250 μl were made as pre-competent cultures, frozen, and stored at -80°C. When transformation of cultures was tested, 200 μl of competent cells were added into 4 ml AGCH medium supplemented with 0.2% sucrose and 0.001% CaCl_2_ and incubated at 30°C, 20 min. After incubation, 1 ml of competent cells received 400 ng of DNA (see Results), and to this mixture, 100 ng of CSP-1 was added (no CSP-1 was added to control cultures). Transformation mixtures were incubated at 30°C, 30 min and then transferred to 37°C, 90 min to allow phenotypic expression. Serial dilutions were made and different amounts of cells were plated on 1% AGCH agar supplemented with 100 μg/ml streptomycin (chromosomal transformants) or not (total cell counts). Plates were incubated 16 h at 37°C, and the number of transformants was determined. These assays were repeated three times.

$$[Transformation\,\;frequencies=\frac{Number\,\;of\,\;transformants}{Total\,\;cell\,\;counts}\times 100$$

### Competence Assays

Competence development was tested by using a transcriptional fusion of the *luc* gene, which encodes luciferase, to the *ssbB* gene that is specifically induced at competence. The *ssbB::luc* fusion reports development of competence by luciferase light emission (Prudhomme and Claverys, 2007). Competence assay were performed as described (Caymaris et al., 2010). Briefly, *S. pneumoniae* strains R6luc and R6ΔPezATluc were inoculated (1:40 dilutions) in C+Y medium (pH 7.0) supplemented with trypsin (2 μg/ml) and allowed to grow at 37°C until OD_550_∼0.2. Cells were harvested and concentrated to OD_550_∼0.8 in fresh medium containing 10% glycerol and kept at -80°C. To monitor the development of spontaneous competence, the cells were thawed and inoculated (1:50 dilutions) in C+Y medium prepared at various pH. For each sample, 182.7 μl of diluted cells were mixed with 13.3 μl firefly D-luciferin (Thermo Fisher Scientific; 10 mM) in a 96-well clear bottom white plate (Corning), followed by incubation at 37°C in a Varioskan Flash Multimode Reader (Thermo Scientific). RLU and OD_492_ were measured every 7 min intervals. Values correspond to individual cultures representative of three independent experiments.

## Results

### The Pneumococcal *pezAT* Operon: Organization, Construction of Deletion Mutant, and Phenotypic Traits

**FIGURE 1:**
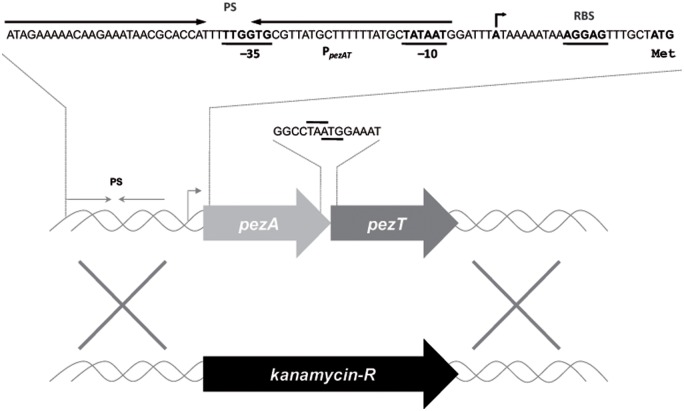
**Schematic organization of the pneumococcal *pezAT* operon.** The scheme depicts the position of the single promoter sequence (line with arrowhead pointing to the direction of transcription), which includes the -35 and -10 regions, as well as the inverted repeated sequence (PS, convergent arrows) that is the binding site of PezA and PezT proteins. Ribosome-binding site sequences are denoted as RBS and the initial start site is also depicted with arrow. The *pezA* termination codon, and the overlapping *pezT* initiation codon are also indicated. Substitution of the entire operon by a gene cassette encoding resistance to kanamycin is shown below.

The genome of *S. pneumoniae* R6*wt* (Tettelin et al., 2001) harbors a single copy of the *pezAT* operon (*spr0951*–*spr0952*; NCBI accession no. NC_003098), located within the PPI1 (Khoo et al., 2007). Other pneumococcal strains, like CGSP14, ATCC 700669, and P1031 have two copies of the same operon (Chan et al., 2012). The presence of the *pezAT* operon has been related to pneumococcal virulence (Brown et al., 2004). Like the typical Type II TAs, the *pezA* antitoxin gene is placed upstream of *pezT* (**Figure 1**), and both genes overlap by one nucleotide, suggestive of coupled translation (Chan et al., 2012). Co-transcription of both genes is directed by a single promoter that is located upstream of *pezA*. This region also includes a long inverted repeat which spans the -35 and -10 regions of the promoter and that is the region where PezA/PezT proteins bind to control their own synthesis: binding of the PezA:PezT to their target would hinder binding of the host RNA polymerase to the promoter, leading to transcriptional repression of the operon (Khoo et al., 2007; Chan et al., 2016). We have studied the influence of *pezAT* on the pneumococcal lifestyle without recurring to previous general approaches that consist of the overexpression of the toxin gene, either in the homologous or in a heterologous host, followed by analysis of the resulting effects (Christensen and Gerdes, 2003; Khoo et al., 2007; Nieto et al., 2007). Instead, we constructed a mutant strain (R6ΔPezAT) in which the entire *pezAT* operon, including the upstream intergenic region was deleted and replaced by a gene encoding kanamycin (**Figure 1**), confirming the observation that the operon is not essential for *S. pneumoniae* (Brown et al., 2001). Under these conditions, lack of the operon could be studied on a number of pneumococcal responses.

### PezAT Modulates Pneumococcal Growth at the Stationary Phase

**FIGURE 2:**
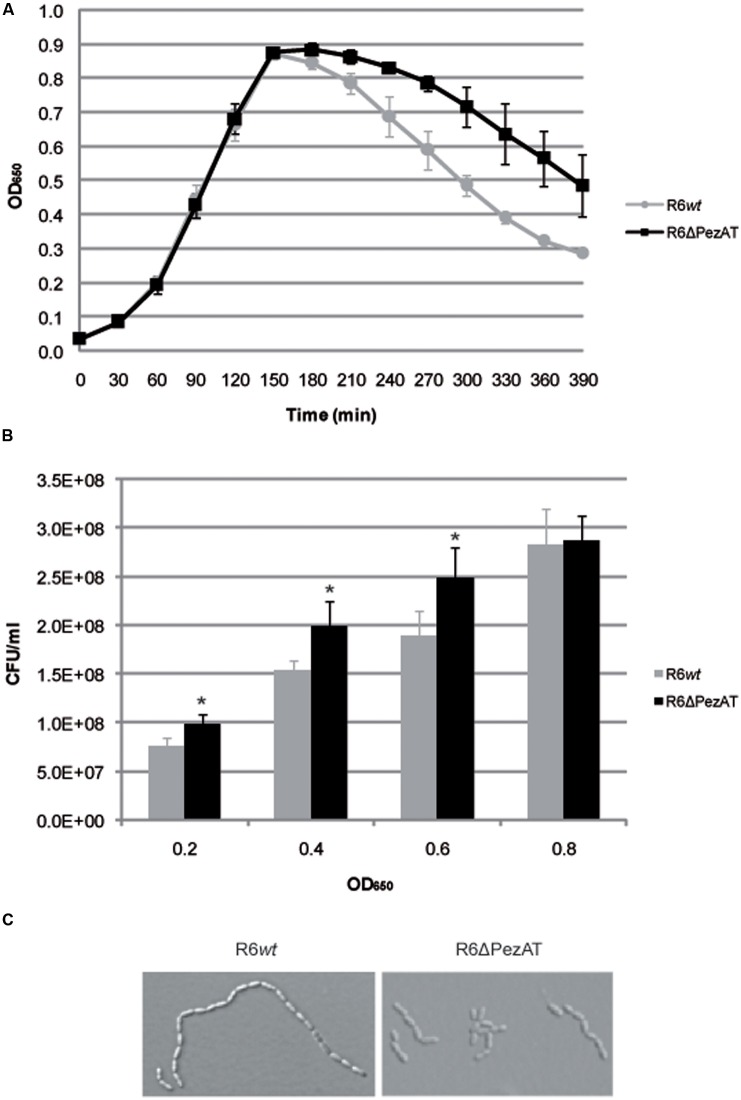
**The *pezAT* mutant strain forms shorter chains and is more prone to lysis at stationary phase.** **(A)** Growth patterns for both R6*wt* and R6ΔPezAT mutant strains were assessed by measuring OD_650_ every 30 min. **(B)** The number of CFU/ml at OD_650_∼0.2, 0.4, 0.6, and 0.8 were determined. *t*-test was used to evaluate the statistical differences and the asterisk symbol (^∗^) denotes statistical significance (*p*-value < 0.05) between R6*wt* and R6ΔPezAT. **(C)** The morphology of the cells of both strains was examined under phase-contrast microscope. Pictures were taken at 100× magnifications, 200 ms.

When the R6*wt* and the R6ΔPezAT strains were tested for growth under normal growth condition (37°C, no aeration, AGCH medium pH7.7), no differences were found at the exponential phase (**Figure 2A**). These observations agree with a previous report (Brown et al., 2004) in which no differences were found in growth in laboratory broth, serum or blood when the *pezT* gene was deleted. In our conditions, however, differences between the two strains were evident when the cultures entered into the stationary phase: the R6ΔPezAT mutant showed a higher resilience to lysis compared to the R6*wt* (**Figure 2A**). We took this finding as a solid indication of the participation of the *pezAT* operon in the pneumococcal growth. Despite no differences in growth patterns during exponential phase for both strains were observed, the CFU counts were lower for the *wt* than for the mutant along the exponential phase, as determined by plating appropriate dilutions of cultures at various ODs and counting of the colony numbers (**Figure 2B**). Given that PezT targets polymerisation of pneumococcal cell walls, it was interesting to know whether this phenomenon was due to changes in the cell morphology. This postulation was assessed by examination of growing cells under phase-contrast microscopy (**Figure 2C**). Indeed, R6ΔPezAT mutant formed shorter chains than the R6*wt* strain at exponential phase. However, the effect was reduced at the beginning of stationary phase, and difficult to assess at prolonged incubation times (OD_650_∼0.8), due to lysis and appearance of ‘ghosts’: cell walls, cell debris, etc. Longer cell chains in the R6*wt* than in the mutant should lead to formation of less CFU in the former than in the latter strains as one colony would result from a string of cells. One possibility could be that the levels of one or more pneumococcal lytic enzymes were slightly increased in the mutant strain. If this were the case, a good candidate would be LytB because, contrary to LytA, it is a non-autolytic murein hydrolase that allows localized peptidoglycan hydrolysis and separates daughter cells (Rico-Lastres et al., 2015); inactivation of *lytB* led to formation of long chains integrated by more than 100 cells (De Las Rivas et al., 2002). Thus, increases in the levels of LytB by the deletion of *pez*T, would result to a reduced number of cells per chain (Johnston et al., 2016). This hypothesis, however, needs further experiments.

Two more straightforward phenotypic tests were also performed: colony appearance to check phase variation (Weiser et al., 1996), and colony sizes and morphologies. However, no significant differences were found between strains R6*wt* and R6ΔPezAT.

### PezAT Does Not Play a Role in Oxidative Stress

**FIGURE 3:**
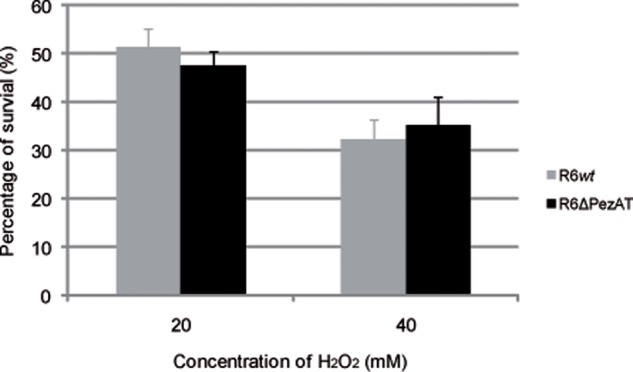
**Oxidative stress affects equally *Streptococcus pneumoniae wt* and Δ*pezAT* strains.** Pneumococcal cultures at OD_650_∼0.3 were treated with H_2_O_2_, 20 min, 37°C, and the number of survival cells were counting to determine the number of CFU/ml on solid medium. *t*-test was used to evaluate the statistical differences and no differences (*p*-value < 0.05) were observed for both strains under oxidative stress.

*Streptococcus pneumoniae* is a facultative anaerobe that colonizes the human nasopharynx of up to 70% of healthy individuals (Chan et al., 2012; Gamez and Hammerschmidt, 2012). As a consequence, the bacteria are customarily exposed to an oxygen-rich environment under colonization conditions, and thus the most frequent stress found by pneumococcal cells *in vivo* is oxidation. Pneumococci also produce H_2_O_2_ in amounts that can exceed 1 mM (which is 1,000-fold higher than the concentration needed to inhibit growth of *E. coli* cells) under aerobic and rich-nutrient conditions. In this way, resident pneumococci will kill or inhibit growth of other respiratory tract-colonizing flora, but growth of *S. pneumoniae* was not impaired even at the high levels of endogenously produced H_2_O_2_ (Pericone et al., 2003). Further, it was shown that pyruvate oxidase, which is the enzyme responsible for production of endogenous H_2_O_2_, also contributed to H_2_O_2_ resistance in pneumococci (Pericone et al., 2003). Although the ability of *S. pneumoniae* to cope with oxidative stress is still not well-understood, we explored whether *pezAT* could be involved in the response to this stress. This was done by exposing R6*wt* and R6ΔPezAT mutant to high concentration of exogenously added H_2_O_2_. The results (**Figure 3**) did not show any significant survival differences between the two strains at any of the tested H_2_O_2_ concentrations (20 and 40 mM), indicating that the *pezAT* operon was not involved in protecting the pneumococcal cells from oxidative stress. Another set of experiments were designed to test whether differences in biofilm formation between the *wt* and the mutant strains could be observed, since it has been shown that some TAs influence biofilm formation (Gonzalez Barrios et al., 2006; Wen et al., 2014).

Biofilm formation was quantified by measuring the OD_595_. The bacterial growth at 37°C was similar in all the tested strains, and no significant differences were found in biofilm formation between the *wt* and the mutant even at 34°C, that is the nasopharynx temperature (**Supplementary Figure S1**).

### PezAT Enhances Sensitivity of *S. pneumoniae* to β-Lactam Antibiotics

**FIGURE 4:**
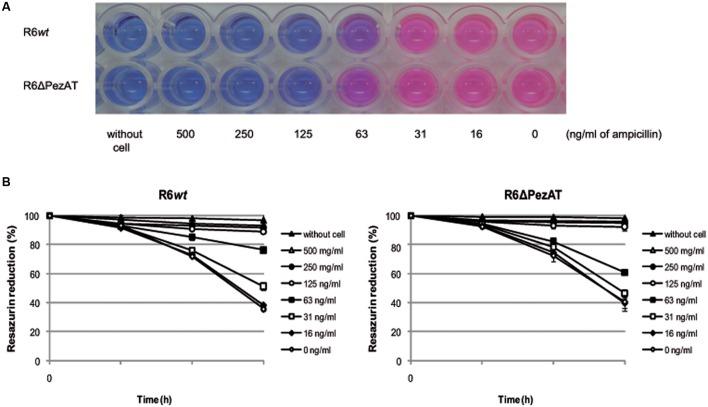
**Deletion of the *pezAT* operon increases resistance to ampicillin.** **(A)** Pneumococcal strain-resistances detected by MIC, determined by change of color from resazurin (blue) to resorufin (pink). **(B)** Color changes measured by light absorbance at 600 nm (resazurin) as a function of the time of incubation, at the different concentrations of ampicillin indicated. Controls: no cells and no antibiotic. *t*-test was used to evaluate the statistical differences (*p*-value < 0.05).

The decreased response to lysis at stationary phase of the *pezAT*-deficient strain (**Figure 2A**), combined with the role of PezT on cell wall synthesis (Mutschler et al., 2011), prompted us to test whether deletion of the *pezAT* influenced the resistance/sensitivity of the pneumococcal cells only to β-lactam antibiotics or it was a more general stress response effect (Nieto et al., 2010). We used REMA plates and color change of resazurin (blue) to resorufin (pink) to compare the MICs of R6*wt* and R6ΔPezAT to antibiotics acting at different levels. The MICs are defined as the lowest concentration of the antibiotic that prevented color change, i.e., no bacterial growth (Andrews, 2001). By examining these changes, we found that the MICs of both strains to ampicillin was similar, 125 ng/ml (**Figure 4A**). However, at 63 ng/ml of ampicillin, the color changes from blue to pink was more prominent for the mutant than for the *wt* strain, indicating that the former strain was more resistant to ampicillin than the latter (**Figure 4A**). This observation was corroborated by measurement of the OD readings in which reduction of resazurin absorbance by the mutant strain (∼40%) was more prominent than the *wt* (∼24%) at 63 ng/ml of ampicillin after 3 h, and the differences were statistically significant (*p*-value < 0.05; **Figure 4B**). Similar results were observed when the MICs were estimated by the dilution method (Andrews, 2001; repeated four times and with a *p*-value < 0.05; not shown). Also similar results were observed for other β-lactam antibiotics, like benzetacil and penicillin G (not shown). No differences between *wt* and mutant strains were found when other antibiotics inhibiting gyrase (levofloxacin), or protein synthesis (streptomycin, tetracycline) were tested (not shown). Taken these results together, we conclude that lack of the *pezAT* operon does not induce a general stress response in *S. pneumoniae*, but that this response in focalized at the cell wall level.

### PezAT Reduces Transformability of *S. pneumoniae*

Natural competence is a state of some bacterial species (the best known being *S. pneumoniae* and *Bacillus subtilis*) that is characterized by two main traits: (i) it is a bistable phenomenon, in which competence is expressed stochastically only in a part of the population, and (ii) the competent cells show growth arrest (Claverys et al., 2006; Hahn et al., 2015). Although, there has not a report showing a direct relationship between TA functionality and transformability, both processes share the two above features (Chan et al., 2012). To assess whether the *pezAT* operon influenced pneumococcal transformability, the two strains, mutant and *wt*, were tested for their transformation frequencies with DNA. To this end, we used competent cultures treated or not with CSP-1, and two different types of DNA. The first one consisted total chromosomal DNA (Chr) isolated from a pneumococcal strain that harbors a point mutation conferring resistance to streptomycin (Lacks, 1966). The second type of DNA (Str41) consisted of a 2002 bp-homogeneous DNA fragment PCR-amplified from Chr with primer pair rpsL_3/rpsL_4 (**Supplementary Table S1**); this fragment also harbors the above point mutation (Caymaris et al., 2010). As controls for genetic complementation, we used the same strains but harboring an ‘empty’ low-copy number plasmid (pC194r) or the same plasmid in which the entire *pezAT* operon (including its own transcription/translation signals) was cloned (pC104rPezAT). Both plasmids confer resistance to chloramphenicol.

**FIGURE 5:**
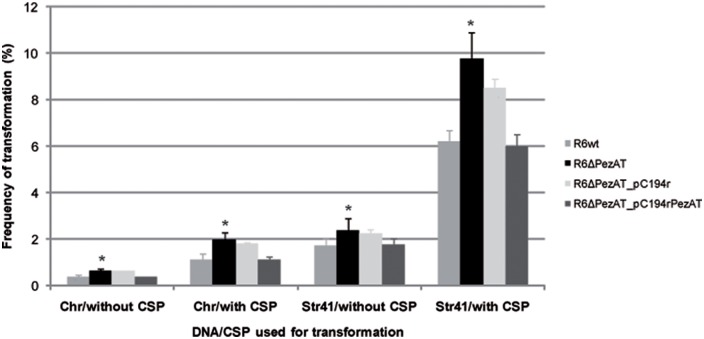
**Deletion of the *pezAT* operon leads to cells with increased transformability.** Competent pneumococcal cells of indicated strains were treated or not with CSP-1 and incubated with transforming DNA prepared from total cell lysates (Chr) or from homogeneous DNA amplified by PCR from total cell lysates (Str41). *t*-test was used to evaluate the statistical differences and the asterisk symbol (^∗^) indicates statistical significance (*p*-value < 0.05) between R6*wt* and R6ΔPezAT.

Under homogenous growing environment and transformation procedures, with Chr DNA, the parental strain R6*wt* showed transformation frequencies of 0.3% in the absence of CSP-1, and increased to 1.1% when CSP-1 was added (**Figure 5**). As expected for homogeneous DNA (López et al., 1982), employment of Str41 DNA led to a substantial increase in the frequencies of transformation: 1.7% (without CSP-1) and 6.2% (with CSP-1; **Figure 5**). In the case of the mutant strain R6ΔPezAT, transformation frequencies augmented significantly (*p*-value < 0.05) ∼1.4- to 1.8-fold compared to the R6*wt* strain at all four different conditions used. Genetic complementation was observed when the strain used carried plasmid pC194rPezAT, but not with the negative control, ‘empty’ plasmid (**Figure 5**).

### PezAT Decreases Competence of *S. pneumoniae*

**FIGURE 6:**
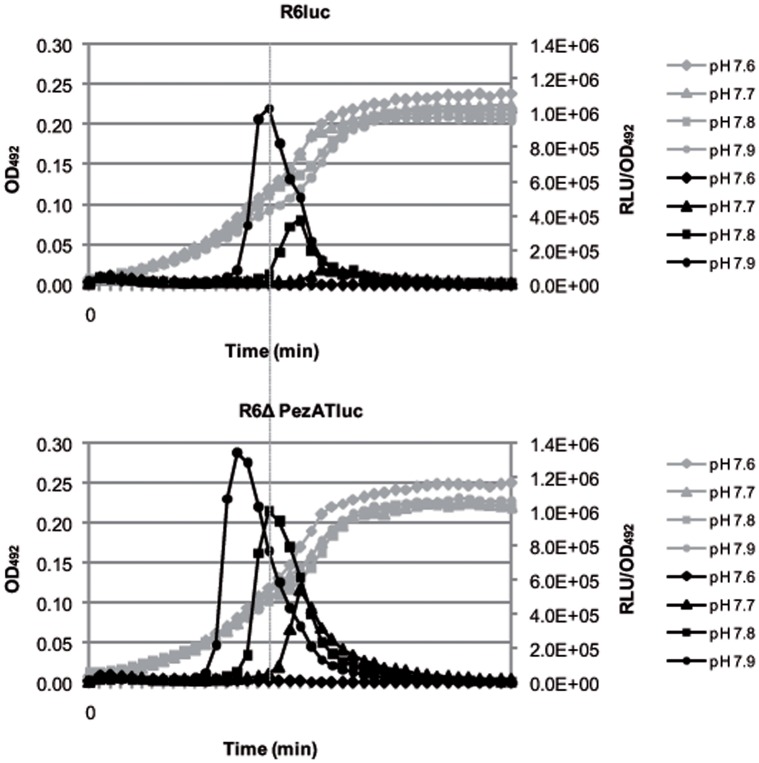
**Deletion of the *pezAT* operon increases genetic competence.** The development of spontaneous competence of both pneumococcal *wt* and *pezAT* mutant cells were assessed by measuring, at different pH, the transcriptional level of *Photinus pyralis luc* gene that was fused downstream of the promoter of *ssbB*, which is a late competent gene. Gray lines indicate growth curves at OD_492_, whereas black lines depict RLUs/OD_492_, ranging from pH 7.7–7.9. Vertical line joining both panels points the time differences in the onset of the main peaks of competence. Values correspond to individual cultures representative of three independent experiments (Caymaris et al., 2010).

The above results showed that the transformability of *S. pneumoniae* increased in the strain devoid of the *pezAT* operon. However, they do not allow us to conclude that genetic competence was different in both strains. Consequently, we made use of the observation that induction of spontaneous competence strongly relies on the initial pH of the cultures (Chen and Morrison, 1987). To determine whether PezAT plays a role in regulation/development of competence, we used a gene cassette with a transcriptional fusion of the luciferase gene to the late competence gene *ssb* (Caymaris et al., 2010). The cassette was inserted into the chromosome of strains R6*wt* and R6ΔPezAT, thus constructing the two isogenic strains R6luc and the mutant R6ΔPezATluc. Media with initial pH ranging from 6.4 to 7.9 were used to investigate the time of occurrence and the level of competence of both strains. Under the same conditions, we observed that both R6*wt* and R6ΔPezAT mutant strains developed spontaneous competence only from pH 7.7 onward, and no prominent development of spontaneous competence was observed from pH 6.4 to 7.6 (only data from pH 7.6 to 7.9 are shown; **Figure 6**). Even though the R6luc and the R6ΔPezATluc strains developed spontaneous competence at the same initial pH (i.e., 7.7), the time of competence development and the magnitude of the competence peaks were different. At initial pH 7.7, the R6luc strain developed spontaneous competence at 140 min and peaked at 154 min with RLU/OD_492_∼8.9 × 10^4^, whereas the R6ΔPezATluc mutant stain started at 112 min and reached the peak at 140 min with ∼6.1-fold higher magnitude. Similar results were observed at medium with initial pH 7.8, where R6luc parent strain started to develop spontaneous competence at 119 min and peaked at 140 min with RLU/OD_492_∼3.8 × 10^5^; whereas the mutant began at 98 min and peaked at 119 min with ∼2.7-fold higher magnitude. For medium with initial pH 7.9, spontaneous competence started for the parent and the mutant strain at 98 and 77 min, respectively; they reached the competence peak at 119 and 98 min, respectively. Further, the ratio RLU/OD_492_ for the mutant was 1.3-fold higher than the parent strain (RLU/OD_492_∼1.0 × 10^6^). In summary, the mutant strain developed spontaneous competence much earlier and with higher magnitude, even though the growth rate for both strains was similar in medium with initial pH 7.7–7.9. Combining all these observations, we conclude that the *pezAT* operon has a significant influence on the competence development and hence, transformability of *S. pneumoniae*.

## Discussion

Toxin–antitoxins constitute an intriguing example of acquisition of genetic information through horizontal gene transfer and manipulation by the host bacterial population to serve as relevant players in their lifestyle. When TAs are located in mobile elements, they ensure their stable inheritance (Hayes, 2003; Guglielmini et al., 2008). However, once located on the bacterial chromosome, they seem to be domesticated to perform functions related to the bacterial physiology, namely response to stressful conditions by causing temporal cell growth arrest, mediators of programmed cell death, or persistence as response to antibiotic challenges (Engelberg-Kulka et al., 2004; Díaz-Orejas et al., 2010; Chan et al., 2012; Gerdes and Maisonneuve, 2012; Gerdes, 2013).

The pneumococcal PezAT TA is harbored by the PPI1 of *S. pneumoniae* R6, which is a mobile element (Brown et al., 2004) and that contributes to strain variations in pneumococcal virulence (Harvey et al., 2011). The PezAT operon is unique in the sense that it is the only known TA that target synthesis of the bacterial cell wall rather than replication or translation (compiled in Gerdes, 2013). We have found that, under normal conditions, PezAT plays a role in the pneumococcal lifestyle which is subtle but evolutionarily relevant. We can assume that the effects of PezAT on growth, CFU formation, resistance to β-lactams, and genetic competence could be due to either triggering of the synthesis of PezT when the cells entered into the stationary phase or by slow leakage and accumulation of PezT toxin due to selective cleavage of the antitoxin PezA along the cell cycle. This second assumption seems more likely to us since degradation of the Epsilon (a homolog of PezA) antitoxin by the host ClpXP protease (Brzozowska and Zielenkiewicz, 2014) would tend to release some of the PezT toxin, albeit in small quantities. It has been shown that Toxin ζ is able to induce reversible dormancy, thus participating in response to stress (Tabone et al., 2014). Leakage of the toxin would lead to small but detectable differences in strains harboring or not the *pezAT* operon: tinkering with the pneumococcal cell wall functionality by low amounts of PezT would lead to early lysis, decreased resistance to β-lactam antibiotics, and slight stress situations that would trigger the onset on competence (**Figures 2**, **4**, and **6**).

How could we, then, envisage whether acquisition of the *pezAT* operon by certain (but not other) pneumococcal strains has any evolutionary advantage? We can propose two, not mutually exclusive, mechanisms. First, acquisition of the *pezAT* operon could be considered as a process of gain of new genetic traits; this matter has been dealt when debating the gain-of-function experiments (Duprex et al., 2015), but the concept should not be limited to that. As noted (Casadevall and Imperiale, 2014), “gain-of-function means exactly what it says, that the entity in question has gained a new property.” In the case of the pneumococcal *pezAT* operon, we can envisage that this gaining could be the horizontally transferred PPI1 acquired by some pneumococcal strains. The second scenario would contemplate a parasitic invasion of PPI1 followed by domestication and use of the newly acquired DNA (Touchon et al., 2014). In either situation, strains that have acquired PezAT would benefit of exhibiting increased virulence. PezAT would ensure that the island is stably inherited (Brown et al., 2004), because cells that lose this particular TA would be killed or displaced from the population by the toxicity of PezT. We can conclude that a trade-off must exist between (i) acquisition of PPI1 (together with its accompanying *pezAT* operon) and hence increased virulence, and (ii) loss-of-function, such as resistance to β-lactam antibiotics. Lysis of the *wt* strain (which is also a virulence trait because of the concomitant release of the Ply citolytic protein; Kadioglu et al., 2008), would be gained when the *pezAT* operon is acquired. However, we should not expect that gain/loss is an all-or-none phenomenon (binary function, bit). On the contrary, we should expect a mild result, since this is the way of evolutionary processes: every little step counts; big strides are not an evolutionary advantage. Evolution works as a continuum made of tiny quanta. We believe we have discovered a novel and natural trade-off process in *S. pneumoniae*, associated to a TA, in which improved colonization and adaptation to their natural niches (exalted virulence) resulted in moderate loss (β-lactam resistance, genetic competence).

## Conclusion

As a difference with previous studies which were performed either in *E. coli* or in *B. subtilis* (Lioy et al., 2006; Khoo et al., 2007; Mutschler et al., 2010), we have used the PezAT natural host and under near physiological conditions, i.e., we have not overproduced the toxin as a mean to analyze their effect. We propose that, in the *wt* cells, PezT toxin accumulates along the cell cycle leading to slight destabilization of the cell wall and, as a consequence, the cells become more prone to lysis. Acquisition of the PPI1, harboring the *pezAT* genes, by the pneumococcal cells would lead to a trade-off between gain of some traits (increased virulence) and loss of some other functions (β-lactam antibiotics resistance and susceptibility to lysis). We predict that the pneumococcal strains that have adopted the PPI1 by horizontal gene transfer would have to give up some resilience to lysis, resistance to β-lactams, and genetic competence, but they would gain the virulence traits carried by the genes of the island.

## Author Contributions

ME and WC designed the experiments, which were performed by WC. Both authors wrote the manuscript.

## Conflict of Interest Statement

The authors declare that the research was conducted in the absence of any commercial or financial relationships that could be construed as a potential conflict of interest.

## Abbreviations

CFU: colony-forming unit
CSP: competence stimulating peptide
MIC: minimum inhibitory concentration
OD: optical density
PPI1: Pneumococcal Pathogenicity Island 1
REMA: resazurin microtiter assay
RLU: relative luminescence unit
TA: toxin–antitoxins
UNAG: uridine diphosphate-*N*-acetylglucosamine
*wt*: *wild type*
