# Supplementary material for: The Streptococcus pneumoniae pezAT Toxin–Antitoxin System Reduces β-Lactam Resistance and Genetic Competence
Source: Front Microbiol. 2016 Aug 25;7:1322. doi: 10.3389/fmicb.2016.01322 (PMC4997998; doi:10.3389/fmicb.2016.01322)
Supplement: Supplementary file 1 [file Data_Sheet_1.DOCX]

**Supplementary Information**

**The *pezAT* toxin-antitoxin genes of *Streptococcus pneumoniae*: Involvement in resistance to β-lactam antibiotics and in genetic competence**

Wai Ting Chan* and Manuel Espinosa*

*Centro de Investigaciones Biológicas, Consejo Superior de Investigaciones Científicas, Ramiro de Maeztu, 9, 28040 Madrid, Spain*

*Correspondence: [mespinosa@cib.csic.es](mailto:mespinosa@cib.csic.es); [chanyting@hotmail.com](mailto:chanyting@hotmail.com)

Supplementary Table S1: Primers used

| Primers | Sequences 5’ 🡪 3’ |  | Restriction sites |
| --- | --- | --- | --- |
| kan-F | AGCAGAGCTCCTTATCGATACCGTCGACCTC |  | *Sac*I |
| kan-R | AGCAACTAGTCCCCTATCTAGCGAACTTTTA |  | *Spe*I |
| pezATup-F | GACGAAGTGATTAAGTATAA |  | - |
| pezATup-R | AGCAGAGCTCATTTATTCTTGTATTAATTT |  | *Sac*I |
| pezATdown-F | AGCAACTAGTACAATTGATATTTTTAGGAG |  | *Spe*I |
| pezATdown-R | CCATTGATTAACCCATGATT |  | - |
| ssbB´luc-F | GATATGGTCATGGTGAAGAC |  | - |
| ssbB´luc-R | TAATTCTATTGTATCACTTG |  | - |
| pezAT-F | CAGCAAGCTTTTATTGTTGAATTTTGGGTA |  | *Hin*dIII |
| pezAT-R | CAGCAAGCTTCCTCTCATTTCTATTCTCCT |  | *Hin*dIII |
| rpsL_3 | TGACATGGATACGGAAGTAG |  | - |
| rpsL_4 | ATGGTAAGCTGAGTTATAGC |  | - |

*Underlined are restriction sites introduced

**FIGURE S1.** Deletion of the *pezAT* operon does not affect pneumococcal biofilm formation. Biofilm formation capacity of the following strains of *S. pneumoniae*R6: *wt*, *wt* harbouring the low copy-number plasmid pC194r, ∆*pezAT*, and ∆*pezAT* harbouring plasmid pC194r in which the *pezAT* operon is cloned. Strains were grown in 96-well polystyrene plates at 34°C, 6 h in C medium. Cells were stained with crystal violet and the plates were rinsed to remove non-attached cells. Grey and black bars indicate growth and biofilm formation, respectively. The values of biofilm formation were normalized for absorbance and the percentages were calculated in relation to the *wt* strain. The results are the average of three independent experiments, and standard error bars are shown.

*3.3. Biofilm formation and quantification*

Biofilm formation assays were performed essentially as previously described (Moscoso et al., 2006). Briefly, pneumococcal strains were grown in C+Y medium to OD_595_ of 0.5–0.6. Cells were sedimented by centrifugation, resuspended and diluted 1:10 in C medium, and dispensed approximately 4.5 × 10^6^ cfu per well in 96-well polystyrene microtiter dishes (Costar 3595, Corning Inc.). After 6 h of incubation at 34°C, the biofilm formed was stained with 0.2% crystal violet and rinsed to remove non-adherent bacteria. After solubilising the biofilm in 95% ethanol, the OD_595_ was determined using a plate reader (Anthos microplate absorbance reader 2020; Anthos Labtec Instruments). The results represent the mean ± standard error of three independent experiments, each of them carried out in triplicate.

REFERENCES

Moscoso, M., García, E., and López, R. (2006). Biofilm formation by *Streptococcus pneumoniae*: role of choline, extracellular DNA, and capsular polysaccharide in microbial accretion. *J. Bacteriol.* 188**,** 7785-7795.
